# Endometrial Staining of CD56 (Uterine Natural Killer), BCL-6, and CD138 (Plasma Cells) Improve Diagnosis and Clinical Pregnancy Outcomes in Unexplained Infertility and Recurrent IVF Failures: Standardization of Diagnosis with Digital Pathology

**DOI:** 10.3390/diagnostics13091557

**Published:** 2023-04-26

**Authors:** Suheyla Ekemen, Cem Comunoglu, Cavit Kerem Kayhan, Ebru Bilir, Ilkay Cavusoglu, Nilay Etiler, Selcuk Bilgi, Umit Ince, Cevayir Coban, Halit Firat Erden

**Affiliations:** 1 Vocational School of Health Services, Kerem Aydınlar Campus, Acıbadem University, Istanbul 34752, Turkey; 2 Acibadem Central Pathology Laboratory, Kerem Aydinlar Campus, Istanbul 34752, Turkey; 3 Department of Pathology, Dr. Cemil Tascioglu City Hospital, University of Health Sciences, Istanbul 34668, Turkey; 4 School of Medicine, Bahcesehir University, Istanbul 34349, Turkey; 5 Women’s Health and Gynecological Nursing, Institute of Health Sciences, Biruni University, Istanbul 34010, Turkey; 6 Department of Public Health, School of Medicine, Istanbul Okan University, Istanbul 34947, Turkey; 7 Public Health Department, University of Nevada, Reno, NV 89509, USA; 8 Department of Digital Pathology, School of Medicine, Acıbadem University, Istanbul 34752, Turkey; 9 Division of Malaria Immunology, Department of Microbiology and Immunology, Institute of Medical Science (IMSUT), University of Tokyo, Tokyo 108-8639, Japan; 10 Obstetrics and Gynecology Infertility Clinic, Zorlu Center, Istanbul 34340, Turkey

**Keywords:** unexplained infertility (UI), recurrent IVF failures, endometriosis, endometritis, CD56 (uNK), BCL-6, CD138

## Abstract

In women with unexplained infertility (UI) and recurrent in vitro fertilization (IVF) failures, the etiology is often unclear. Endometrial immune perturbations and the use of immune markers associated with these dysregulations are of great interest in the diagnosis and treatment of UI. However, reliable biomarkers and standardized quantification methods are lacking. Here, to address endometrial immune dysregulation in UI patients with recurrent IVF failures, we performed endometrial tissue sampling and immunostaining of CD56 (uNK), CD138, and BCL-6. Of these cases, 57.9% had positive CD56 in the endometrial stroma, while 46.1% had positive BCL-6 in the glandular epithelium, and 14.5% of the cases were found to be positive for CD138. Combined staining rates were 60.5%, 68.4%, and 71.05% for (CD56 or BCL-6), (CD56 or CD138), and (CD56, BCL-6, or CD138), respectively. There was a significant correlation between CD56 and BCL-6 positivity, while CD138 positivity was an independent parameter. After the recommended targeted therapy, pregnancy rates were found to increase from 58.5% to 61.6% and 73.8% in CD56-positive, (CD56- or BCL-6-positive), and (CD56-, BCL-6-, or CD138-positive) cases, respectively. Notably, a retrospective evaluation of digital pathology and light microscopy results showed a significant correlation. This study suggests that the examination of CD56, BCL-6, and CD138 in the same endometrial sample may be an effective method in determining the etiology of UI and reaching an early diagnosis and treatment options. Moreover, digital pathology can be used in the evaluation of CD56 and BCL-6 to provide objective, rapid, and reliable results.

## 1. Introduction

Unexplained infertility (UI) is defined after normal results of intensive infertility evaluation and accounts for approximately 15–30% of infertility cases [1]. In vitro fertilization (IVF) is used in cases of UI; however, recurrent IVF failures are common [2]. Endometriosis is thought to be among the significant causes of UI. However, laparoscopic diagnosis of endometriosis may not be accurate [3], and accurate diagnosis is important for successful treatment outcomes [3,4]. In recent years, the use of immune markers that are related to changes in endometrial immunity and microbiota and the detection of immune disruption of the endometrium have received considerable attention in the diagnosis, staging, and treatment of UI [5,6,7]. However, there are no standardized criteria on how to objectively assess and validate these immune markers in the endometrium.

The leukocytes in the endometrium are clearly different from those of the peripheral blood and consist mainly of uterine natural killer cells (uNK, ~70%) and other cells such as macrophages, neutrophils, mast cells, dendritic cells, and T and B cells [6,7]. The uterine NK cells express CD56 (the uNK marker, also known as the neural cell adhesion molecule), but not other classical NK cells or T-cell markers. The number of uNK cells is known to change during the menstrual cycle, pregnancy, and various pathologies of the endometrium, with controversial results. Activated uNK cells are involved in the regulation of trophoblast invasion into the decidua [8,9]. The elevation of CD56, which normally shows a slight increase in the endometrial stroma during the preimplantation period, has been found to be higher in infertile women and in pregnancy loss [2,8,10,11,12]. There appears to be a direct correlation between CD56 elevation and pelvic endometriosis [11,13,14,15,16,17,18,19]. 

B-cell lymphoma 6 protein (BCL-6) was first identified as an oncogene important for proliferation in B-cell lymphomas but was later found in various tumors, as well as endometrial pathologies. Several recent studies have investigated the relationship between the expression rate of BCL-6 in the endometrial gland epithelium and endometriosis [5,20,21,22,23]. Increased BCL-6 expression has been shown in UI and recurrent IVF failures [21,22]. BCL-6 overexpression has been associated with increased cellular proliferation [24]. A high expression of BCL-6 in women with endometriosis has been associated with a decrease in progesterone activating and regulating receptors and the inactivation of progesterone in the endometrium [22]. BCL-6 is thought to be responsible for progesterone resistance in the endometrium of women with endometriosis [20,22,23]. BCL-6 is also the master regulatory gene that is essential for T follicular helper cell differentiation, as well as B cell differentiation in germinal centers (GCs), and its regulation by various factors may play a role in autoantibody development [25,26]. Therefore, an abnormal expression of endometrial BCL-6 may be responsible for poor reproductive outcomes after embryo transfer [21,27].

Chronic endometritis is an inflammation of the endometrium that prevents implantation and is another possible cause of infertility [7]. The immunohistochemical presence of plasma cells (CD138, also known as syndecan-1) in endometrial specimens is thought to be an objective diagnosis of chronic endometritis [28]. However, there is still no clear consensus on the threshold for the number of plasma cells in the endometrium and its correlation with UI, and the treatment outcomes have not been confirmed.

We hypothesized that a combination of different immune markers would be a better diagnostic approach in the endometrial material from women with UI and recurrent implantation failures. Therefore, here, we evaluated the combination of three markers (CD56, BCL-6, and CD138) in curettage material from women with UI and recurrent IVF failures. We examined the association between these biomarkers and clinical pregnancy outcomes after treatment. Digital image analysis has recently been introduced for cell density estimation of the endometrium as an easier, faster, and standardized assessment of pathology specimens [17,29,30]. Therefore, we additionally compared immunohistochemical panel assessment with light microscopy (LM) and digital pathology (DP) performed by expert pathologists in order to assess and validate the objective criteria without major diagnostic errors and to eliminate any differences between the assessments.

## 2. Materials and Methods

### 2.1. Study Population and Ethics Approval

In this retrospective study, the endometrial biopsy materials of 76 women with UI and recurrent IVF failures were included. The inclusion criteria were as follows: (i) no male infertility, (ii) no evidence of endometriosis by laparoscopic or clinical examination, (iii) no laboratory evidence of acute infections, such as HCV, HBV, HIV, or Rubella, and (iv) no abnormal serum prolactin, T3, T4, FSH, LH, Estradiol (E2), and progesterone (P4) levels. The women were aged 23–45 years (33.7± 4.5, mean ± SD), had no previous pregnancies, and had experienced at least one, and up to 8, IVF failures (Table 1). After IVF, all of the women were routinely followed up for successful clinical pregnancy outcomes with serum beta-hCG (10–11 days after implantation) and ultrasound (presence of gestational sac and the fetal heartbeat investigated 4–5 weeks after implantation). A total of 65.8% of the women were aged 30–39 years, and 57.9% had 3 or more IVF failures. A total of 15.8% of the women had 5–8 IVF failures, while more than 80% had 1–4 IVF failures. 

Ethical approval was obtained from Acibadem University Faculty of Medicine Ethics Committee (ATADEK2019-1/14).

### 2.2. Endometrial Sampling and Immunostaining 

Endometrial sampling was performed in the secretory phase between days 22 and 23 of the cycle after daily monitoring of endometrial thickness by ultrasound and blood hormone levels every other day. All samples were fixed in 10% neutral-buffered formalin solution and processed with a Tissue-Tek Vip^®^ 6 AI device (Sakura Finetek Japan Co., Ltd., Tokyo, Japan) [31] to prepare paraffin blocks. Three-µm-thick sections were prepared from all of the blocks and stained with hematoxylin and eosin (H&E) using a Shandon Gemini stainer. Immunohistochemical staining was performed using antibodies against CD56 (CD564 clone, Leica Biosystems, Wetzlar, Germany), CD138 (B-A38 clone, Biocare Medical, Pacheco, CA, USA), and BCL-6 (LN 22 clone, Biocare Medical, Pacheco, CA, USA) using a Ventana Benchmark XT device (Roche Diagnostics, Basel, Switzerland). 

### 2.3. Evaluation by Conventional Light Microscopy (LM)

All of the slides were morphologically evaluated and reported by two independent pathologists (S.E. and C.Com.) who were experienced in the field of gynecological pathology using a light microscope (LM) (Olympus BX51). CD56+ cells in the stroma were assessed on a semiquantitative scale as a percentage of the endometrial stromal cells, where a reference value of more than 6% was established (<6% is considered negative and ≥6% is considered positive staining). For CD138 staining, two independent pathologists screened the entire slide (~100 × 80 mm^2^ area) and the entire stroma section at ×400 magnification, and ≥1 plasma cell was considered to be a positive value [32]. The number of CD138-positive plasma cells ranged from 7 to 121 cells (53.7 ± 41.4, mean ± SD) in positive cases. BCL-6+ cells in the glandular region were assessed using the proposed HSCORE (0–4) system to minimize the difference between the observers [5,33]. Briefly, the HSCORE was calculated as the membranous staining intensity of BCL-6 in glandular cells (absent: 0, weak: 1, moderate: 2, and strong: 3) and the epsilon value was obtained by dividing the sum of the product of the individual gland percentages by 100, with the formula HSCORE = ∑ Pi (I + 1)/100 (i = staining intensities and Pi = the percentage of stained cells for each intensity, ranging from 0% to 100%) [5]. An HSCORE reference value of ≥1.4 was considered positive staining and <1.4 was considered to be a negative BCL-6 value. Neuroendocrine tumor sections for CD56 and lymph node sections for BCL-6 and CD138 were used as positive controls.

### 2.4. Digital Pathology (DP)

In addition to the conventional pathology reports, the same slides were re-evaluated with the 3DHISTECH CaseViewer program (3DHISTECH Ltd., The Digital Pathology Company, Budapest, Hungary), a digital microscopy application designed to support the histopathologic diagnosis and microscopy review process at Acibadem University, which has been used since 2014 [34]. As with conventional light microscopy, CD56 positivity was calculated as a percentage in stromal cells. BCL-6 staining in the glandular epithelium was, to our knowledge, digitally calculated for the first time in this study using the HSCORE (0–4) scoring system. Since we considered ≥1 plasma cell positivity with LM as positive-CD138 staining, and a sign of chronic endometritis, we did not evaluate CD138 positivity with DP separately. 

### 2.5. Statistical Analysis

All statistical analyses were performed with IBM SPSS (Statistical Package for the Social Sciences) version 28 (IBM Corp., Armonk, NY, USA). Associations between continuous data were tested using Pearson’s correlation tests. In all analyses, the statistical significance level (*p*-value) was considered to be less than 0.05.

## 3. Results

### 3.1. Immunopathology Detection of the Endometrium by Conventional Light Microscopy

**Figure 1:**
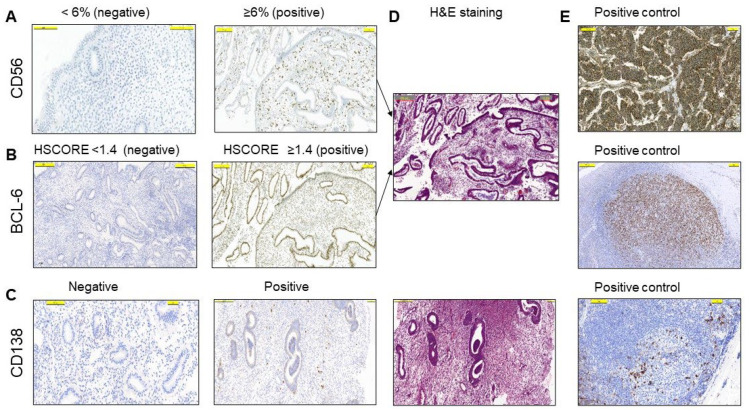
The semiquantitative analysis of the endometrium by conventional light microscopy (LM). (**A**) Representative examples of positive (≥6%) and negative (<6%) cells with immunohistochemical staining for CD56 in the stroma. (**B**) Representative examples of HSCORE grading of positive (≥1.4) and negative (<1.4) cells with immunohistochemical staining for BCL-6 in the glandular epithelium. (**C**) Representative examples of CD138 positivity and negativity in the stroma. (**D**) H&E staining of corresponding positive IHC sections in (**A**–**C**). (**E**) Irrelevant neuroendocrine tumor section in A and lymph node sections in B and C were used as positive controls. The pictures are at the same magnification.

A total of 76 women aged 23–45 years (33.7 ± 4.5, mean ± SD) with at least one, and up to eight, IVF failures were included in this study after careful exclusion of other factors, such as male infertility, infections, endometriosis, and hormonal abnormalities (Table 1). CD56 positivity in the endometrial stroma by LM was calculated as <6% (negative) and ≥6% (positive) (Figure 1A). A total of 44 (57.9%) cases had values of 6% or more for CD56 immunostaining in the endometrial stroma, while 35 cases (46%) had an HSCORE of 1.4 or higher for BCL-6 immunostaining in the glandular epithelium (Figure 1B and Table 2), and 11 cases (14.5%) were positive for CD138 staining and were considered to be chronic endometritis (Figure 1C). Corresponding H&E sections and positive controls are shown in Figure 1D,E, respectively. 

Two or more IVF failures occurred in 74.3% of CD56+ cases and 65.7% of BCL-6+ cases. It is noteworthy that 75% of those CD56+ cases (33 out of 44) had BCL-6 HSCORE values above 1.4 in the glandular epithelium. Pearson’s correlation test revealed a moderate but significant correlation between CD56 and BCL-6 positivity, with r = 0.576 and *p* < 0.001 (Table 3). In contrast, among the CD138+ cases, CD56 positivity was observed in only two cases (18.2%) and BCL-6 positivity in only one case (9%). Therefore, we considered CD138 positivity as an independent, but additional, endometrial marker for the immune pathology of the endometrium, i.e., chronic endometritis. On the other hand, when combined, CD56 and BCL-6 positivity increased to 60.5%, while the overall detection of endometrial pathology with the combination of CD56, BCL-6, and CD138 was 71.05% (Table 2). Twenty-two cases (28.9%) did not show positive immunostaining with any marker.

### 3.2. Targeted Treatment Based on Immune Marker Detection Increased Pregnancy Rates

After examining the cases with immunohistochemistry, the infertility center was advised to use CD56 positivity ≥6% and BCL-6 HSCORE ≥ 1.4 as references for the treatment options based on the previous literature [5,17]. When the CD56 reference value exceeded the positivity criteria (≥6%), the patients were treated with cortisone and/or intralipid therapy [16,35]. A high BCL-6 HSCORE is generally used to detect both occult endometriosis and the development of progesterone resistance [20]. However, due to the higher levels of BCL-6 and CD56 positivity together, 94.3% of the BCL-6+ cases were treated similarly to the CD56+ cases, except for one patient who refused treatment despite double positivity with BCL-6 and CD56. In addition, any positive value for CD138 staining in the endometrial stroma was considered to be chronic endometritis and received antibiotic treatment [36]. The cases without positivity for any of the studied markers were evaluated for other options.

Finally, we followed the patients for clinical pregnancy success and compared them with the immune marker results in the pathology report. Clinical pregnancy was routinely assessed by monitoring serum beta-hCG levels and ultrasonographic evaluation for the presence of a gestational sac and fetal heartbeat. Overall, successful pregnancy outcomes were achieved in 85.5% of cases (65 out of 76 cases). We then evaluated the contribution of single and/or combined immunostaining results to successful clinical pregnancy outcomes. Single positive staining with CD56, BCL-6, or CD138 showed relatively higher clinical pregnancy rates (58.5%, 46.2%, and 16.9%, respectively). However, the highest pregnancy success occurred in the total cluster of cases staining positive for CD56, BCL-6, or CD138 markers, with 73.8% of clinical pregnancies occurring in this group (Table 2). On the other hand, the group in which all of the markers were negative (comprising 28.9% of all cases) contributed only 26.5% of the successful clinical pregnancy rates. Together, these results suggest that increased numbers of immune marker detection, followed by targeted therapy, significantly improve clinical pregnancy rates.

### 3.3. Digital Pathology Analysis of CD56 and BCL-6 Immunostaining of the Endometrium

**Figure 2:**
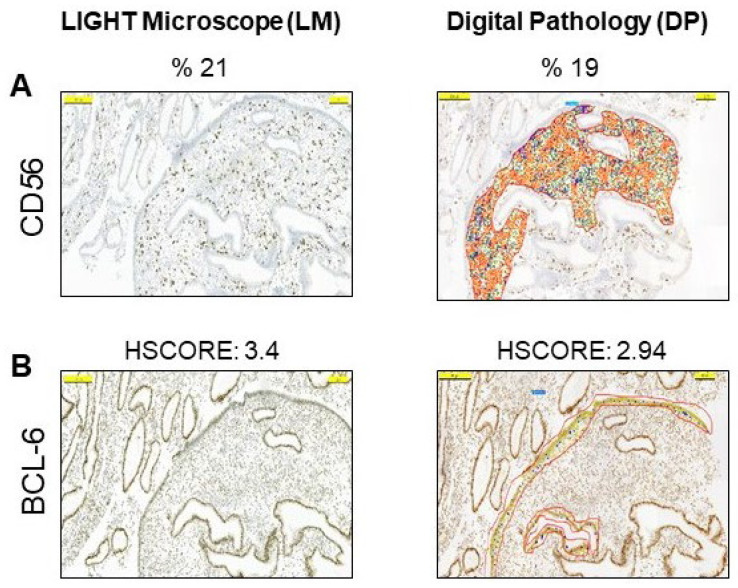
Digital pathology analysis of the endometrium by 3DHISTECH CaseViewer. (**A**) Representative examples of positive cells with digital imaging for CD56 in the stroma. (**B**) Representative examples of HSCORE grading of positive cells with digital imaging for BCL-6 in glandular epithelium. The pictures are at the same magnification.

We then evaluated the same slides using the DP setting of the 3DHISTECH CaseViewer program, which has been installed, optimized, and routinely used at Acibadem University since 2014 [34]. The criteria for CD56 or BCL-6 positivity did not differ from the conventional microscopy evaluation methodology, but this time the evaluation was performed with DP (Figure 2A). Similar to the LM results, DP yielded values of 6% or higher for CD56 immunostaining in the endometrial stroma in 44 cases (57.9%), and HSCORE values of 1.4 or higher for BCL-6 immunostaining in the glandular epithelium in 35 cases (46%) (Figure 2B).

To investigate the value of DP over conventional microscopy, we compared the LM assessment results of both CD56 and BCL-6 with the DP results. There was a statistically strong correlation between the CD56 values measured by LM and DP (r = 0.90, *p* < 0.001, by Pearson’s correlation test). There was also a high correlation between the HSCORE values of the BCL-6 assessed by LM and the BCL-6 assessed by DP (r = 0.94, *p* < 0.001, by Pearson’s correlation test) (Table 3). In addition, a significant correlation between CD56 and BCL-6 positivity with both LM and DP (r = 0.576, *p* < 0.001 and r = 0.592, *p* < 0.001, respectively, by Pearson correlation test) was observed (Table 3). Furthermore, when the LM results of the CD138 levels were added, the percentage of endometrial pathology diagnosed by DP reached 69.7%, comparable to the LM analysis. 

## 4. Discussion

Endometrial immune dysregulation is well recognized as a cause of UI and recurrent IVF failure; however, there is no consensus on which biomarkers or standard diagnostic criteria should be evaluated. Here, we found that a combination of immunohistochemical staining of the endometrial stroma with uNK cell marker CD56 and the plasma cell marker CD138, together with BCL-6 immunostaining of the glandular epithelium in sections of the same specimen, can significantly improve the diagnostic and therapeutic outcomes of UI and IVF failures. Notably, the evaluation of three different immunostains can be reliably standardized using digital pathology, which enables the quick, easy, and objective evaluation of specimens.

Previous studies have shown that, apart from physiological endometrial cycles [2], CD56 levels in the endometrial stroma can also be elevated when immunological disturbances occur in the endometrium, and recurrent implantation failures may occur due to cytokine release from these CD56+ uNK cells [2,11,12,13,17,18,37]. In this study, we found that the CD56 levels were highly elevated in the endometrial stroma of most women (57.9%) with UI and recurrent IVF failure. Although there is no clear reference value for determining CD56 levels [10], the use of cortisone and/or intravenous intralipid has been recommended when CD56 positivity is above 6%, based on the previous reports [16,38]. Therefore, the infertility center was guided to set the reference value as ≥6% CD56 positivity for the treatment decision and follow-up. According to the LM review by two independent pathologists, 44 cases with high CD56 levels were treated with cortisone and/or intralipid, and pregnancy occurred in 86.4% of these cases. Although calculating the percentage of CD56 positivity in the endometrial stroma with LM gives clear results, it is laborious and time consuming. Moreover, since the assessment is subjective, there may be variations between the evaluators. Our findings revealed that DP of immunopathology slides, as a new technology [39], showed a strong correlation with LM results (Table 3). Therefore, it should be considered that CD56 immunostaining can be objectively, reliably, and rapidly assessed by DP in the future.

Immunohistochemical staining of BCL-6 in the endometrium has recently emerged as a new marker to be evaluated when the cause of infertility is in question [5,20,21,22,23]. Increased staining and intensity of BCL-6 in endometrial glands has previously been associated with the identification of pelvic endometriosis^5,^ where it may interfere with embryo implantation [20,22]. Therefore, the measurement of BCL-6 levels in the endometrial tissue is valuable, both for the definitive detection of endometriosis^3^ and for possible progesterone resistance in the endometrium [20]. We evaluated BCL-6 levels by LM based on HSCORE scoring and found 46% positivity in our cases. We also performed, to the best of our knowledge, the first digital assessment of BCL-6 immunostaining based on HSCORE and found that DP and LM results showed a strong correlation (Table 3); however, there were also some unexpected differences. For example, in one case, although the LM assessment value was very low (HSCORE = 0.5), the digital pathology measurement was 1.6. In this case, the CD56 level in the stromal cells was calculated as 4% (below the cut-off value of <6%), therefore, the case did not receive treatment and pregnancy did not occur. Would the outcome have been different if this case had received treatment according to the high BCL-6 level that was calculated by DP? We do not know the answer to this. However, this example shows that BCL-6 assessment in LM can vary greatly depending on the interpreter. Therefore, it seems that HSCORE assessment of BCL-6 with digital pathology may be more reliable and faster.

CD138 immunostaining was positive in 14.5% of the cases, while only two cases were found to be positive with CD56 immunostaining, suggesting that there is no correlation between CD56 and CD138 positivity. Our evaluation showed that the LM assessment setting of ≥1% for CD138 positivity did not require DP evaluation. Therefore, we did not perform CD138 assessment by DP, and strongly recommend screening of immunohistochemistry sections by LM for CD138 evaluation. Nevertheless, the positive cases that were diagnosed with chronic endometritis were treated with standard antibiotic therapy [28]. Surprisingly, all 11 cases achieved pregnancy after such treatment. Although a few studies disagree with our findings [32], we recommend that CD138 immunostaining be considered as an independent marker for the evaluation of endometrial specimens. However, a larger sample size is needed in the future for a more definitive decision. 

Our analysis of two and three staining procedures in the same sample showed that the percentage of endometrial perturbation diagnosis in all of the cases increased by 57.9%, 60.5%, and 71.05% for CD56, CD56 or BCL-6, and CD56, BCL-6, or CD138 positivity, respectively. Interestingly, among the immune-marker-positive cases (all but one of the case received treatment), the pregnancy rate reached 73.8% for the three-marker-stains (CD56, BCL-6, or CD138). Three-marker-negative staining was seen in only 28.9% of cases, and the contribution of this group to the successful clinical pregnancy rate was only 26.15%, well below the marker-positive and treated group. Given the limited number of cases in this group, larger studies with appropriate controls are still needed in the future for a definitive analysis. 

In summary, we suggest that CD56, CD138, and BCL-6 immunomarkers should be studied together in a single session in curettage material from women with UI and recurrent IVF failure in the same cycle. In specimens with all three immunohistochemical stains, chronic endometritis (CD138) and CD56 elevation (an increase in uNK cells) can be detected first, and a specific treatment can be easily given. The secondary benefit is directed towards BCL-6. In our study, BCL-6 correlated well with CD56 positivity, even better than CD56 immunopositivity alone. In addition, since BCL-6 positivity is associated with pelvic endometriosis, immunostaining of curettage material may allow for an easy diagnosis and protect individuals from more invasive interventions. Therefore, further studies are needed in order to evaluate BCL-6 positivity in the endometrium. 

## 5. Study Limitations

The current study lacks further interpretation due to the lack of a control group. However, our recommendation is to examine these three markers in recurrent IVF failures after UI and to use digital pathology to provide more objective quantitative data when evaluating CD56 and BCL-6.

## Figures and Tables

**Table 1:** Characteristics of the study population who were diagnosed with unexplained infertility (UI).

Characteristics	Number (*n* = 76)	Percentage (%)
**Age groups**		
<25	1	1.3
25–29	17	22.4
30–34	25	32.9
35–39	25	32.9
≥40	8	10.5
**Number of IVF failures**		
1–2	32	40.8
3–4	32	40.8
5–6	10	15.8
7–8	2	2.6

**Table 2:** The rates of clinical pregnancy outcome by immune markers.

Immune Markers	Definition	Positivity Rate *n* = 76 (%)	Clinical Pregnancy Outcome *n* = 65 (%)
CD56 Levels	≥6%	44 (57.9)	38 (58.5%)
BCL-6 Levels	HSCORE ≥ 1.4	35 (46.1)	30 (46.2%)
CD56 or BCL-6	CD56 ≥ 6% or BCL-6 HSCORE ≥ 1.4	46 (60.5)	40 (61.5%)
CD56 or CD138	CD56 ≥ 6% or CD138 ≥ 1	52 (68.4)	46 (70.8%)
CD56, BCL-6, or CD138	CD56 ≥ 6%, BCL-6 HSCORE ≥ 1.4, or CD138 ≥ 1	54 (71.05)	48 (73.8%)

**Table 3:** Correlations of CD56 and BCL-6 immunohistochemical markers with light microscopy (LM) and digital pathology (DP).

	Correlation Coefficient (r)	*p* *
CD56 (LM) vs. CD56 (DP)	0.906	<0.001
BCL-6 (LM) vs. BCL-6 (DP)	0.943	<0.001
CD56 (LM) vs. BCL-6 (LM)	0.576	<0.001
CD56 (DP) vs. BCL-6 (DP)	0.592	<0.001

LM: Light microscopy, DP: Digital pathology. * Pearson’s correlation test.

## Data Availability

The data that support the findings of this study are available at a reasonable request from the corresponding author, S.E.

## Abbreviations

BCL-6	B-cell lymphoma 6 protein
CD138	Plasma cell marker, also known as Syndecan-1
DP	Digital pathology
IVF	In vitro fertilization
LM	Light microscopy
UI	Unexplained infertility
uNK	Uterine natural killer
